# Catheter-Directed Thrombolysis for Postpartum Deep Venous Thrombosis

**DOI:** 10.3389/fcvm.2022.814057

**Published:** 2022-04-26

**Authors:** Miguel Girona, Christoph Säly, Vladimir Makaloski, Iris Baumgartner, Marc Schindewolf

**Affiliations:** ^1^Division of Angiology, Swiss Cardiovascular Center, University Hospital of Bern (Inselspital), University of Bern, Bern, Switzerland; ^2^Department of Medicine I, Academic Teaching Hospital Feldkirch, Feldkirch, Austria; ^3^Department of Cardiovascular Surgery, Swiss Cardiovascular Center, University Hospital of Bern (Inselspital), University of Bern, Bern, Switzerland

**Keywords:** venous thromboembolism, iliofemoral deep vein thrombosis, catheter-directed thrombolysis, pregnancy, postpartum

## Abstract

Venous thromboembolism is a major concern during pregnancy as well as in the postpartum period. In acute proximal deep venous thrombosis, endovascular recanalization with locally administered thrombolytic agents has evolved as therapeutic alternative to anticoagulation alone. However, data on the bleeding risk of thrombolysis in the postpartum period is limited. We addressed the key clinical question of safety outcomes of catheter-directed thrombolysis (CDT) in the peri- and postpartum period. Therefore, we performed a non-exhaustive literature review and illustrated the delicate management of a patient with postpartum acute iliofemoral thrombosis treated with CDT and endovascular revascularization with thrombectomy, balloon angioplasty and stenting.

## Introduction

Venous thromboembolism is a major concern during pregnancy and the postpartum period and represents the main cause of mortality during the postpartum in developed countries (1). The risk of venous thromboembolism is fivefold increased during pregnancy and up to 60-fold in the postpartum period (2, 3). The annual incidence amounts to 200 cases per 100,000 women (4).

The thrombophilic condition during pregnancy and postpartum period is mainly the result of a hormone-related hypercoagulability with elevated concentrations of factors VII, VIII, and X, fibrinogen and von Willebrand factor. In addition, intrinsic thrombolytic capacity is decreased due to higher levels of plasminogen activator inhibitor (5). These hemostatic changes occur in preparation of delivery and are supposed to be protective against peripartum hemorrhages. However, these prothrombotic changes persist for several weeks after delivery. Besides these systemic risk factors for VTE, local factors impairing venous inflow play an important role, in particular venous compression by the growing fetus (6). These local factors explain in part the higher rate of proximal deep venous thrombosis (DVT) during late stage pregnancy.

Proximal DVT is associated with a high morbidity. Postthrombotic syndrome (PTS) represents the most important long-term complication of DVT that is associated with a significant reduction in the quality of life. As many as 50% of patients with iliofemoral DVT develop a severe PTS (7).

To prevent PTS after acute proximal DVT, catheter directed thrombolysis (CDT), i.e., endovascular recanalization with locally administered thrombolytic agents, which is followed by mechanical thrombectomy and stent placement in most of the cases, has been suggested as a therapeutic alternative to anticoagulation alone (8, 9). However, bleeding complications are a major adverse effect of any thrombolysis and must be weighed against the benefits of quick clot removal (10). There is paucity of data on thrombolysis after DVT in the high risk constellation of postpartum patients. Therefore, we addressed the key clinical question of safety outcomes of CDT in the peri- and postpartum period and performed a non-exhaustive literature review.

## Literature Review

We performed a review of the literature on CDT in the postpartum period, applying the following search terms in the PubMed database: [(peripartum OR postpartum OR pregnancy) AND thrombolysis], without any further search limits. After the primary query, the obtained abstracts were screened manually whether deep vein thrombosis was the underlying clinical condition and which complications and which other therapies, e.g., thrombectomy, stenting, were applied.

## Results

**FIGURE 1:**
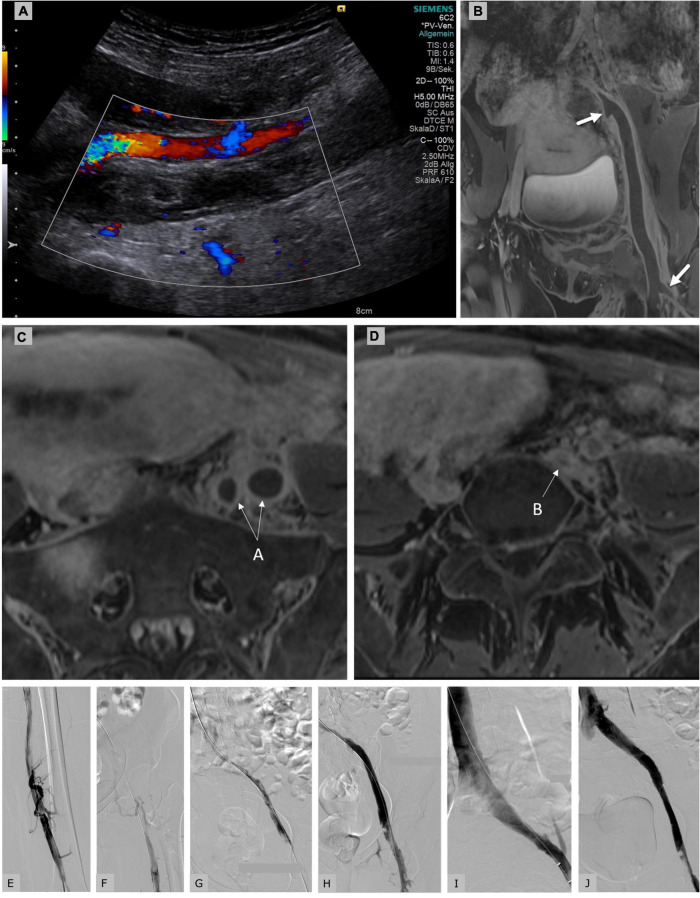
Duplex sonography **(A)** and MR-phlebography **(B)** of a 31-year-old female patient 10 days postpartum showing an iliofemoral thrombosis with involvement of the internal iliac vein (proximal arrow) and the deep femoral vein (distal arrow) down to the left popliteal vein. Clinically the left leg was tender and cool with slight sensible, but no motoric impairment; imminent phlegmasia was suspected. The patient was placed on a therapeutic dose of intravenously administered unfractionated heparin (Liquemin^®^, Drossapharm, Basel, Switzerland) which was adjusted according to repeated aPTT measurements. In the absence of clinical signs of bleeding and with a hemoglobin level within the normal range, catheter-directed thrombolysis (CDT) was performed via transcutaneous access of left occluded popliteal vein. After a bolus of 10 mg, a continuous infusion of alteplase (Actilyse^®^, Boehringer Ingelheim, Basel, Switzerland) was initiated at 2 mg/h for 5 h, and then reduced to 1 mg/h for 10 h. A total dose of 30 mg alteplase was applied. Insertion of an inferior vena cava filter was rejected after interdisciplinary discussion. Breast-feeding was paused for 24 h, but breast milk was collected before CDT to be fed to the infant later. aPTT levels were always documented to be within the therapeutic range (64–85 s) that is 1.5–2.5 times more than the baseline aPTT of 30 s. After discontinuation of thrombolysis the patient was placed on anticoagulation with subcutaneously administered enoxaparin 0.9 mg/kg bid (Clexane^®^, Sanofi, Vernier, Switzerland). **(C)** MR-phlebography of the same patient showing the transversal view of the enlarged thrombosed external and internal iliac veins (arrow A). **(D)** Proximal common iliac vein compression in the context of May-Thurner syndrome (arrow B). Initial phlebography of the partially occluded femoral veins **(E)** and of the occluded iliac veins **(F)** showing fresh thrombus. Control venography of our patient after catheter-directed thrombolysis **(G)**, after Angiojet^®^ thrombectomy [Angiojet Zelante^®^ (8F), Boston Scientific, Larlborough, United States] **(H)**, after balloon angioplasty **(I)**, after stenting (Sinus obliquus 16/100, Optimed^®^, Ettlingen, Germany) of the left common iliac vein which was compressed in the context of underlying May-Thurner syndrome with residual thrombosis in the common femoral vein **(J)**. The patient was discharged under anticoagulant treatment with enoxaparin 1.7 mg/kg qd for 3 months. No bleeding complications occurred neither during thrombolysis, pharmacomechanical thrombectomy nor during anticoagulation. No venous thrombembolic events occurred. Stent patency was confirmed sonographically after 3 months. After 4 months of treatment, the patient showed no signs or symptoms of PTS (Villalta-Score 0 point) and identical leg circumferences.

We found 26 reports on single patients or small patient series, totaling 31 patients who had been treated with CDT in the postpartum period. No larger patient series and no randomized controlled trials were available. The mean age of the patients treated was 26.9 years. The time after delivery when thrombolysis was performed varied between 2 and 42 days. Only in one case, thrombolysis was already started during pregnancy (9th gestational week) but the pregnancy was terminated after the treatment. The most frequently used thrombolytic agent was alteplase (15 cases), followed by urokinase (12 cases), and streptokinase (5 cases). Duration of the thrombolytic therapy was agent-dependent and varied from 20 to 30 h for alteplase, 16 to 72 h for urokinase, and 106 to 140 h for streptokinase, respectively. For alteplase the dose was 0.01 mg/kg/h (4 cases), 0.02 mg/kg/h (4 cases), and 1 mg/h (6 cases). For streptokinase and urokinase, mostly 100,000 U/h were used (only in two cases the dosage was slightly higher). A simultaneous unfractionated heparin treatment was routinely administered during thrombolysis except for 4 cases where this information on adjunctive anticoagulation was missing. Of 31 patients analyzed, 22 were treated successfully (<30% residual luminal area narrowing), three patients were treated partially successful. In 6 cases the outcome was not described. Eighteen patients underwent balloon angioplasty with stent placement in 10 cases. Three patients were exclusively treated with thrombolytic therapy. In 10 patients adjunctive therapies besides thrombolysis were not described. Criteria for/against angioplasty and/or stenting were mostly not described. When described, the most reason for angioplasty was stenosis and for stenting was the presence of a May Thurner Syndrom. In one case, an early re-thrombosis occurred (after angioplasty plus stenting). Minor bleedings occurred in 5 cases only and no major or life-threatening bleeding complications were registered. Other complications as anemia, thrombocytopenia, and hemolysis were described casually but no life threatening situations occurred. In more than half of our reviewed patients, no information on PTS was documented. Nevertheless, in the remaining 15 patients, 14 did not develop PTS subsequently, and only one patient showed signs of mild PTS in the further course. Results of our literature review are summarized in Table 1 (11–19). Additionally we illustrated the delicate management of a patient with postpartum acute iliofemoral thrombosis treated with CDT in Figure 1.

**TABLE 1:** Summary of literature review.

					Thrombolytic agent									
Patient	References	Age	Mode of delivery	Time of thrombolysis (days after delivery)	Agent	Bolus	Dosis	Duration (h)	Anticoagulation	Additional intervention	IVC filter insertion prior CDT	Outcome	Complications	PTS
1	(11)	Mean 30 (28–33)	Cesarean	<42	Alteplase	5 mg	0.01 mg/kg/h	20–24	UFH	NS	No	Successful	None	NS
2	(11)	Mean 30 (28–33)	Vaginal	<42	Alteplase	5 mg	0.01 mg/kg/h	20–24	UFH	NS	No	Successful	None	NS
3	(11)	Mean 30 (28–33)	Vaginal	<42	Alteplase	5 mg	0.01 mg/kg/h	20–24	UFH	NS	No	Successful	None	NS
4	(11)	Mean 30 (28–33)	Vaginal	<42	Alteplase	5 mg	0.01 mg/kg/h	20–24	UFH	NS	No	Successful	None	NS
5	(19)	24	Vaginal	8	Streptokinase	NS	100,000 U/h	120	UFH	PTA	No	Partial successful	None	Mild PTS at 6 Mo
6	(19)	22	Vaginal	7	Streptokinase	NS	100,000 U/h	140	UFH	PTA	Yes	Successful	Minor bleeding	No PTS at 6 Mo
7	(19)	20	Vaginal	7	Streptokinase	NS	100,000 U/h	110	UFH	PTA	No	Successful	Minor bleeding	No PTS at 6 Mo
8	(19)	23	Vaginal	10	Streptokinase/Urokinase	NS	100,000 U/h	120	UFH	PTA	No	Successful	Minor bleeding	No PTS at 6 Mo
9	(19)	29	Abortion (Second trimester)	10	Streptokinase	NS	100,000 U/h	120	UFH	PTA	Yes	Successful	Minor bleeding	No PTS at 6 Mo
10	(16)	26	vaginal	14	Alteplase	5 mg	0.02 mg/kg/h	20	NS	None	No	Successful	None	NS
11–16	(14)	Mean 28 (21–42)	NS	<42	Alteplase	NS	1 mg/h	mean 30	UFH	NS	1 of 6 yes	NS	None	NS
17	(15)	26	Vaginal	14	Alteplase	5 mg	0.02 mg/kg/h	22	NS	None	No	Successful	None	NS
18	(15)	34	No information	35	Alteplase	5 mg	0.02 mg/kg/h	23	NS	None	No	Successful	None	NS
19	(15)	30	Vaginal	47	Alteplase	5 mg	0.02 mg/kg/h	24	NS	PTA and Stent	No	Successful	None	NS
20	(13)	24	Cesarean	20	Alteplase	NS	NS	18	UFH	PTA and Stent	Yes	Successful	None	NS
21	(12)	35	Vaginal	3	Urokinase	300,000 U	100,000 U/h	31	UFH	PTA and Stent	Yes	Successful	No major, calf hematoma	No PTS at 16 Mo
22	(12)	22	Vaginal	28	Urokinase	300,000 U	100,000 U/h	49	UFH	PTA and Stent	Yes	70% patency	No major, early rethrombosis	No PTS at 12 Mo
23	(12)	30	Vaginal	14	Urokinase	300,000 U	100,000 U/h	16	UFH	PTA and Stent	Yes	Successful	None	No PTS at 39 Mo
24	(12)	26	No information	21	Urokinase	300,000 U	100,000 U/h	26	UFH	PTA	Yes	Successful	No major, hemolysis	No PTS at 20 Mo
25	(12)	27	Cesarean	42	Urokinase	300,000 U	100,000 U/h	72	UFH	PTA and Stent	Yes	Successful	None	No PTS at 56 Mo
26	(12)	21	Vaginal	11	Urokinase	300,000 U	100,000 U/h	24	UFH	PTA and Stent	Yes	Successful	No major, embolism trapped by filter	No PTS at 18 Mo
27	(12)	27	Cesarean	12	Urokinase	300,000 U	100,000 U/h	48	UFH	PTA and Stent	Yes	Successful	None	No PTS at 26 Mo
28	(12)	23	Vaginal	14	Urokinase	300,000 U	100,000 U/h	27	UFH	PTA and Stent	Yes	Successful	No major, early rethrombosis	No PTS at 12 Mo
29	(12)	29	Cesarean	14	Urokinase	300,000 U	100,000 U/h	46	UFH	PTA and Stent	Yes	70% patency	None	No PTS at 14 Mo
30	(18)	22	Vaginal	15	Streptokinase	NS	150,000 U/h	106	UFH	Thrombus Aspiration/PTA	Yes	Successful	No attributable to thrombolysis	NS
31	(17)	30	Vaginal (twins)	2	Urokinase	NS	137,500 U/h	48	UFH	PTA	No	Successful	No major, minor bleeding	NS

*GW, Gestational week; NS, not specified; IVC, inferior vena cava; REF, reference; PTS, postthrombotic syndrom.*

## Discussion

The aim of CDT therapy in proximal DVT is to restore venous patency in imminent phlegmasia as soon as possible, to preserve arterial perfusion, to attenuate symptoms, to prevent local thrombosis progression and pulmonary embolism and to preserve valvular function in order to reduce the risk of PTS. In case of May-Thurner stenosis as an additional thrombotic risk factor besides the transient risk of hormonal changes during pregnancy and the postpartum period, anticoagulation may be discontinued after 3 months of anticoagulation if May-Thurner lesion was successfully treated by stent placement and an unrestricted venous inflow has been documented before discontinuation of anticoagulation.

There is paucity of data on safety regarding systemic or local thrombolysis in pregnancy or in the postpartum period. Several guidelines consider thrombolysis during pregnancy as a relative but not as an absolute contraindication, recommendations regarding the postpartum period are not specified (20–22). Among the most relevant adverse effects during any thrombolysis are hemorrhagic complications. While the complication rate of systemic thrombolytic therapy does not seem to be higher in pregnant compared to non-pregnant women (major and minor bleeding complications have a rate of 8% each), (23) the major bleeding risk was much greater in the postpartum (58%) than in the antepartum period (18%) (24). The difference is due to the recent delivery or cesarean section and most postpartum bleedings presented as vaginal or intraabdominal hemorrhages with the highest bleeding risk for cesarean section (24, 25). Measures to control postpartum bleedings comprise manual uterus compression, intrauterine tamponade, uterotonic drugs, blood transfusions, and endovascular interventions, e.g., uterine artery embolization, laparotomy, hysterectomy, and recombinant factor VIIa (25).

When performing low-doses of locally administered thrombolytics with CDT in the postpartum period, there is a major concern with an increased bleeding risk from the uterus. It is known that CDT plus anticoagulation confers a significant increase in the occurrence of major bleeding events compared to anticoagulation alone (26). Our literature research revealed 31 published cases, none of whom suffered severe bleeding complications. In order to minimize bleeding risk, it should be respected that the incidence of bleeding complications falls with increasing time interval between delivery and start of thrombolysis (25). Regarding hemostatic management, it is important to consider that global coagulation assays, e.g., aPTT-measurements may be confounded by thrombolytic therapy and changes of coagulation factors in pregnancy and postpartum (5, 27–31). In that case, use of a chromogenic anti-factor Xa assay instead, which provides more stable and predictable therapy adjustment should be considered (32–35). Fibrinogen measurement during alteplase thrombolysis is recommended because bleeding events have been significantly associated with the percent reduction in fibrinogen and have a likelihood ration of 1.4 when fibrinogen level is < 150 mg/dl (36).

However, if bleeding risk is deemed to be too high by the treating physician, single-session mechanical thrombectomy may represent a viable strategy that avoids thrombolytic therapy and its incurring bleeding risk completely and warrants quick restoration of venous inflow (37).

Vena cava filter (VCF) placement should be subject to interdisciplinary discussion because the incidence of symptomatic pulmonary embolism (PE) during CDT is low (38, 39) and the benefit of VCF placement before CDT remains unclear (39–41) and it is currently not standard practice (42). Furthermore, VCF use had no associated benefit regarding in-hospital mortality and contrariwise showed an increased incidence of procedure-related hematoma and a prolongation of hospital stay (42). A current meta-analysis on efficacy and safety of VCF placement for prevention of PE showed a reduction in the number of subsequent PE by 50% and an increase of the risk subsequent DVT by 70%. PE-related and all-cause mortality had no significant benefit (38).

Breast-feeding is another important consideration in this clinical scenario. It is known that alteplase does not cross the placenta because of its molecular size of 59 kDa (43), but it remains uncertain whether alteplase passes into breast milk in humans. However, absorption is unlikely because alteplase is destroyed in the infant’s gastrointestinal tract (43). The short half-life of 3.5–5.0 min explain why we pragmatically decided to pause breast-feeding for 24 h.

As has been shown in a recent meta-analysis, percutaneous endovenous intervention in conjunction with anticoagulation is associated with a significant reduction in PTS, a lower rate of venous obstruction and a lower rate of recurrent DVT (26). Conflicting results of the recently completed Acute Venous Thrombosis: Thrombus Removal with Adjunctive Catheter-Directed Thrombolysis (ATTRACT) trial on CDT in deep vein thrombosis that showed no statistically significant difference in PTS between patients with anticoagulation plus CDT vs. patients with anticoagulation alone (44) must be interpreted with caution. ATTRACT was limited by a high rate of ascending femoropopliteal thromboses, a high rate of implanted not dedicated venous stents, the lack of clear stenting criteria and a lower overall stenting rate which may have hampered the results (45). In patients with an extended iliofemoral thrombosis, imminent phlegmasia and May Thurner syndrome the availability of specifically configured reinforced bifurcational venous stents, may provide good clinical results with prevention of future PTS. However, it must be acknowledged that the reviewed case reports are subject to selection bias, which limits their generalizability.

In conclusion, CDT together with catheter-directed thrombectomy and additional stenting of residual stenotic lesions is effective and safe in postpartum patients with iliofemoral DVT. So far, encouraging case reports from the literature have not documented major bleeding complications during CDT > 2 days postpartum. To definitely clarify this issue larger case series and eventually randomized controlled trials appear necessary.

## Author Contributions

All authors listed have made a substantial, direct, and intellectual contribution to the work, and approved it for publication.

## Conflict of Interest

The authors declare that the research was conducted in the absence of any commercial or financial relationships that could be construed as a potential conflict of interest.

## Publisher’s Note

All claims expressed in this article are solely those of the authors and do not necessarily represent those of their affiliated organizations, or those of the publisher, the editors and the reviewers. Any product that may be evaluated in this article, or claim that may be made by its manufacturer, is not guaranteed or endorsed by the publisher.
