# Erratum: Catheter-directed thrombolysis for postpartum deep venous thrombosis

**DOI:** 10.3389/fcvm.2023.1187149

**Published:** 2023-03-27

**Authors:** Frontiers Production Office

**Affiliations:** Frontiers Media SA, Lausanne, Switzerland

**Keywords:** venous thromboembolism, iliofemoral deep vein thrombosis, catheter-directed thrombolysis, pregnancy, postpartum

An Erratum on Catheter-directed thrombolysis for postpartum deep venous thrombosis By Girona M, Säly C, Makaloski V, Baumgartner I and Schindewolf M. (2022) Front. Cardiovasc. Med. 9:814057. doi: 10.3389/fcvm.2022.814057

An omission to the funding section of the original article was made in error. The following sentence has been added: “Open access funding was provided by the University of Bern”.

The original version of this article has been updated.

